# Molecular investigation of primary aldosteronism: exploring genetic heterogeneity in understudied populations

**DOI:** 10.20945/2359-4292-2025-0228

**Published:** 2025-10-28

**Authors:** Leonardo K. Maeda, Livia M. Mermejo, Fabio L. Fernandes-Rosa, Ayrton C. Moreira, Sonir R. Antonini, Margaret de Castro

**Affiliations:** 1 Departamento de Clínica Médica, Faculdade de Medicina de Ribeirão Preto, Universidade de São Paulo, Ribeirão Preto, SP, Brasil; 2 INSERM, PARCC, Université de Paris, Paris, France; 3 Departamento de Pediatria, Faculdade de Medicina de Ribeirão Preto, Universidade de São Paulo, Ribeirão Preto, SP, Brasil

**Keywords:** Primary aldosteronism, APA, adrenalectomy, pathogenic variations in channel-encoding genes

## Abstract

**Objective:**

Genetic abnormalities in ion channels that regulate the depolarization of adrenal glomerular cell plasma membranes have been identified as a cause of primary aldosteronism (PA) due to aldosterone-producing adenoma (APA). This study aimed to evaluate somatic variants in the *KCNJ5*, *CACNA1D*, *CLCN2*, A*TP1A1*, *ATP2B3*, *GNAQ*, *GNA11*, and *CTNNB1* genes, assess the genotype-phenotype correlation, and analyze the outcomes in patients with APA from a heterogenic ethnic population.

**Subjects and methods:**

Clinical, biochemical, and molecular data were obtained from 32 patients.

**Results:**

Pathogenic variants (PVs) were identified in 43.7% (14/32) of the patients. PVs occurred in 31.2% (10/32) of the *KCNJ5* gene: p.Leu168Arg (15.6%), p.Gly151Arg (9.3%), p.Glu145Gln (3.2%), and p.Gly151_Tyr152del (3.2%). In the *CLCN2* gene, two PVs (6.25%), p.Pro48Arg and p.Ala195Thr, were identified; the latter was found in association with p.Glu145Gln in the *KCNJ5* gene within the same APA. Additionally, two PVs were found in ATPase genes: p.Leu104Arg in *ATP1A1* (3.2%) and p.Leu425_Val426del in ATP2B3 (3.2%). No PVs were identified in the other examined genes. Patients with *KCNJ5* PVs were predominantly female (90% *vs*. 45.5%; *p* = 0.01), had an earlier age of PA diagnosis (38 *vs.* 54 years; *p* = 0.04), and exhibited fewer electrocardiogram abnormalities (20% *vs.* 59%; *p* = 0.04). Patients with PVs across all studied genes also showed an earlier age at PA diagnosis (*p* = 0.02). The Primary Aldosteronism Surgical Outcome score revealed that 37.5% of patients met clinical/biochemical cure criteria, 12.5% showed partial improvement in both, while 50% achieved complete biochemical but not clinical remission. Patients carrying PVs had a higher rate of complete clinical and biochemical cure (66.7% *vs.* 33.3%; *p* = 0.05).

**Conclusion:**

Identifying PVs in this study enhances our understanding of the genetic landscape in Brazilian patients with primary aldosteronism.

## INTRODUCTION

Primary aldosteronism (PA), characterized by dysregulation of aldosterone production despite suppression of plasma renin, is recognized as the most common cause of secondary hypertension (^1^). It remains underdiagnosed (^2^), particularly in underdeveloped countries, leading to variations in its reported prevalence across global centers (^3^). Despite this, recognition of PA has increased since its initial description six decades ago (^3,4^), with its prevalence ranging from 5%-10% among hypertensive patients to 15%-26% in those with resistant hypertension (^5-7^). Aldosterone-producing adenoma (APA) and idiopathic bilateral adrenal hyperplasia are the primary causes of autonomous aldosterone hypersecretion, with carcinoma being a rare etiology. In patients exhibiting adrenal nodules larger than 4 cm, carcinoma should be suspected. Another uncommon cause of PA is unilateral hyperplasia in the adrenal glomerular zone (^8^). Suspected cases of hyperaldosteronism, following screening by elevated plasma aldosterone and suppressed plasma renin, should undergo confirmatory testing, including computed tomography and bilateral adrenal vein sampling (AVS). These diagnostic measures are crucial for differentiating between unilateral and bilateral forms of PA and for guiding appropriate treatment (^5,8^). Aldosterone-producing adenoma is predominantly treated through laparoscopic adrenalectomy, whereas bilateral hyperplasia is typically managed with mineralocorticoid receptor antagonists with or without additional antihypertensive medications (6,8,9).

Follow-up for patients is essential to evaluate the efficacy of either surgical or clinical treatment for PA (^10^). In cases of unilateral APA, the overproduction of aldosterone chronically suppresses renal renin release and reduces aldosterone secretion by both the adjacent non-neoplastic adrenal glomerular zone and the contralateral adrenal gland (^11-13^). Although recommendations for PA diagnosis are well-established, evaluation of outcomes post-adrenalectomy for APA has been limited. However, the Primary Aldosteronism Surgical Outcomes (PASO) study recently established international criteria for assessing both clinical and biochemical treatment success in unilateral APA patients following adrenalectomy (^9,14-16^).

Numerous genetic abnormalities have been identified in both sporadic and familial forms of APA, including alterations in ion channels that regulate the depolarization of adrenal glomerular cell plasma membranes, thereby affecting aldosterone secretion (^11,17^). The most common modifications involve heterozygous somatic variants in the *KCNJ5* gene (^18^), followed by pathogenic variants (PVs) in the *CACNA1H* gene (^19^). Additional heterozygous somatic variants in the *CLCN2* gene and in *ATP1A1* and *ATP2B3* have been described in families with PA participating in a European multicentric study (^20-23^). Activating somatic PVs in exon 3 of the *CTNNB1* gene were identified in 5% of aldosterone-producing tumors, with these variants typically being exclusive. Notably, exceptions occur with β-catenin mutations, where concurrent β-catenin and *KCNJ5* or *CACNA1D* mutations have been infrequently observed. Meanwhile, *GNA11* or \*GNAQ* somatic variants are seen as co-drivers in the pathogenesis of \*CTNNB1-*mutated APAs during periods such as puberty, pregnancy, and menopause (^24-26^).

Vilela and cols. (^27^) conducted the first extensive study investigating somatic PVs in the *KCNJ5*, *ATP1A1*, \*ATP2B3*, and \*CTNNB1* genes in a Brazilian cohort of 76 APAs. These variants were found in 51% (39/76) of the tumors, with *KCNJ5* mutations present in 43% (33/76). In patients with bilateral hyperplasia, the same Brazilian team discovered rare heterozygous germline variants in the *PDE2A* and *PDE3B* genes, with functional studies indicating their pathogenicity (^28^). Further research is required to explore PVs in these and other genes involved in the molecular pathogenesis of PA in Brazilian cohorts. In this study, we investigated somatic variants in genes encoding ion channels (*KCNJ5*, *ATP1A1*, *ATP2B3*, and *CACNA1D*); the *CTNNB1* gene, which encodes β-catenin, a crucial effector of the Wnt/β-catenin signaling pathway; and the *GNA11* and *GNAQ* genes, coding for components of the Gαq/11 signaling pathway that mediates the downstream effects of GPCR-coupled phospholipase Cactivation. We also assessed the genotype-phenotype correlation and the clinical and biochemical outcomes post-adrenalectomy, in accordance with the PASO criteria.

## SUBJECTS AND METHODS

Our cohort consisted of 32 patients with APA who were followed at the University Hospital of Ribeirao Preto Medical School, University of Sao Paulo, Brazil. The study was approved by the Institution’s Ethics Committee, and all participants provided informed consent (protocol nos. 7534/2010 and 1586/2020).

Clinical and laboratory data were collected from the patients’ medical records. Clinical diagnosis of PA and its etiological differentiation were conducted in accordance with the Endocrine Society’s clinical practice guidelines (^5,7^). Following screening and confirmatory tests, all patients underwent a fine-slice CT protocol of the adrenal glands, and among them, 10 also underwent AVS. The remainder did not present a clinical indication for AVS (patients with hypertension before age 40, severe primary aldosteronism characterized by aldosterone levels > 20 ng/dL, suppressed renin, and hypokalemia), demonstrating unequivocal unilateral lesions (>1 cm) without thickening in the contralateral adrenal gland. All but one patient exhibited unilateral aldosterone production, confirmed either by AVS (lateralization index > 4) or via histopathological study following unilateral laparoscopic adrenalectomy. Only one patient exhibited APA in both adrenal glands and subsequently underwent bilateral laparoscopic adrenalectomy. The criteria for biochemical and clinical success following adrenalectomy for unilateral APA were adopted from the multicenter PASO study (^9^).

During surgery, tumor samples were collected. A portion of the tissue was allocated for routine histopathological examination, while another part was immediately frozen, later microdissected to isolate tumor tissue for molecular studies. Tumoral DNA was isolated using a commercial kit (QIAamp DNA Mini Kit, QUIGEN GmbH, Germany), with sample integrity assessed by spectrophotometry at an absorbance of 260/280 nm using NanoDropTM 2000/2000c (Thermo Fisher Scientific, USA) and by agarose gel electrophoresis. PCR was employed to amplify tumoral DNA, using primers for the hotspots of *KCNJ5* (exon 2), \*CACNA1D* (exons 6, 8, 14, 16, 23, 27, and 32), \*CLCN2* (exons 2, 5, 10, 11, and 24), \*ATP1A1* (exons 4 and 8), \*ATP2B3* (exon 10), \*GNAQ* (exon 5), \*GNA11* (exon 5), and \*CTNNB1 *(exons 2, 3, and 4), as described elsewhere (^11,17,23,25^). The amplified products were sequenced using a commercial kit (Big Dye Terminator Cycle Sequencing, Applied Biosystems/Thermo Fisher Scientific, USA), and the obtained sequences were aligned to reference sequences using the CodonCode Aligner V4.0.4TM.

Statistical analysis was performed with GraphPad Prism 8 software (v. 8.02 for Windows). Continuous data were presented as mean, median, and range. Continuous numerical variables were analyzed with the Mann-Whitney U test, whereas the chi-square test (χ²) was utilized for the analysis of categorical variables. A significance level of 0.05 was considered.

## RESULTS

**Figure 1:**
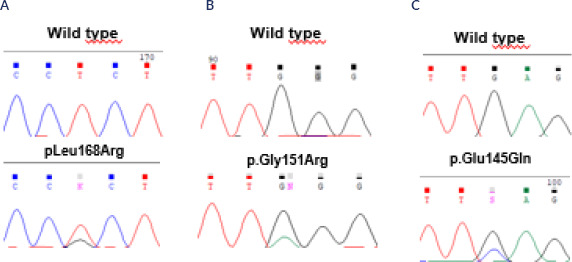
Electropherograms obtained by Sanger sequencing method from three patients with aldosterone-producing adenoma presenting the pathogenic variants (*A*) p.Leu168Arg, (*B*) p.Gly151Arg, and (*C*) p.Glu145Gln in the *KCNJ5*.

**Figure 2:**
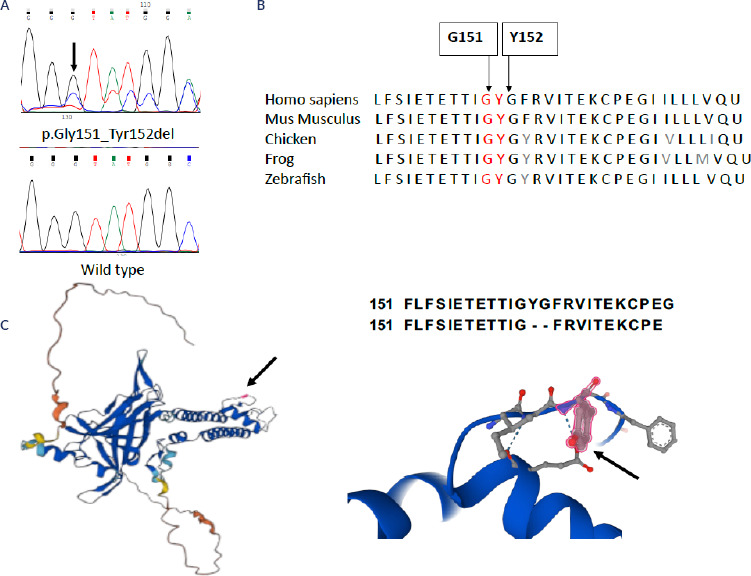
(**A**) Sanger sequencing chromatogram of one patient with aldosterone-producing adenoma showing the novel somatic heterozygous *KCNJ5* variant, which consists of a 6 bp in-frame deletion (c.453_458delGTATGG) resulting in an in-frame deletion of two amino acids, glycine 151 and tyrosine 152, in the protein sequence (p.Gly151_Tyr152del), without altering the downstream reading frame (top), compared to the wild-type sequence (bottom). (**B**) Multiple sequence alignment demonstrating the evolutionary conservation of residues G151 and Y152 in the KCNJ5 protein across different species. (**C**) Predicted 3D structure of the KCNJ5 protein highlighting the location of the deleted residues G151 and Y152 (indicated by arrows), which correspond to the first glycine and the tyrosine of the highly conserved GYG motif within the K^⁺^ channel selectivity filter.

**Table 1** presents the clinical and biochemical data of 32 patients diagnosed with APA, whereas **Table 2** provides a summary of the molecular findings and the frequency of distinct somatic PVs identified in the *KCNJ5*, \*CACNA1D*, \*CLCN2*, \*ATP1A1*, \*ATP2B3*, and *CTNNB1* genes among the 32 patients (33 APAs). We detected PVs in 43.7% (14/32) of the APAs. The most prevalent PVs were found in the \*KCNJ5* gene, with a prevalence of 31.2% (10/32). Notably, the most commonly documented PVs in the literature, such as p.Leu168Arg, were identified in 5 patients (15.6%) (**Figure 1A**), and p.Gly151Arg in 3 patients (9.3%) (**Figure 1B**). Notably, in the patient with bilateral APA, the p.Gly151Arg variant was present in both lesions. Furthermore, the p.Glu145Gln variant was identified in 1 patient (3.2%) (**Figure 1C**). Additionally, in one patient (3.2%), we noted a 6 bp in-frame deletion (c.453_458delGTATGG) in *KCNJ5*, resulting in an in-frame deletion of two amino acids, glycine 151 and tyrosine 152, within the protein sequence (p.Gly151_Tyr152del), without affecting the downstream reading frame (**Figure 2A**). The G151 and Y152 residues in the KCNJ5 protein are highly conserved across multiple species (**Figure 2B**). The structural model of the KCNJ5 protein details the positions of the deleted residues G151 and Y152 (indicated by arrows), corresponding to the initial glycine and the central tyrosine of the highly conserved GYG motif that constitutes the K^⁺^ channel selectivity filter (**Figure 2C**).

**Table 1:** Clinical and biochemical findings of patients with aldosterone-producing adenoma

	All APA patients (n=32)	Patients with pathogenic variants (n=13)	Patients with no pathogenic variants (n=19)	P
Sex (F:M)	19:13	**77%**	**23%**	**0,09**
BMI < 25 kg/m²	22.5%	28.4%	26.3%	0.46
Age at onset of hypertension (years)	35 (17-56)	35 (20-56)	31 (17-40)	0.16
Age at diagnosis (years)	47.5 (27-69)	**38 (27-64)**	**55 (33-69)**	**0.02**
Preoperative DDD	5.7 (0.67-11.67)	4.7 (0.67-11)	6.1 (2.5-11.6)	0.57
Positive family history of hypertension/early cardiovascular disease	53.1%	61.54%	54.55%	0.68
Preoperative MAP (mmHg)	111.7 (80-163.3)	108 (86.6-163.3)	113.3 (80-156.6)	0.43
Potassium (mmol/L)	2.7 (1.7-4.1)	2.6 (2.1-4.1)	2.7 (1.7-4)	0.50
Aldosterone (ng/dL)	46 (13.3-242)	41.8 (14.9-94)	64.1 (13.3-242)	0.32
Renin (mU/L)	2 (<2-3.9)	2 (<2-3.1)	2 (<2-3.9)	0.96
Preoperative creatinine (mg/dL)	0.95 (0.6-2.7)	1.15 (0.6-2.7)	1.1 (0.6-2)	0.36
Preoperative CKD-EPI (mg/dL)	90.5 (18-133)	101.5 (18-120)	88.5 (36-133)	0.65
Normal ECG	53%	69%	42%	0.13
Nodule size (cm)	1.5 (0.6-7)	1.9 (0.6-3)	1.4 (0.6-7)	0.47
Postoperative DDD	1.83 (0-6.83)	1.7 (0-5.3)	2 (0-6.8)	0.96
Postoperative creatinine (mg/dL)	1 (0.6-12)	1 (0.6-3.3)	1 (0.7-12)	0.33

Abbreviations: APA: aldosterone-producing adenoma; BMI: body mass index; DDD: defined daily dose; ECG: electrocardiogram; CKD-EPI: Chronic Kidney Disease Epidemiology Collaboration; MAP: mean arterial pressure. For statistical purposes, renin levels < 2 mU/L were considered equal to 2 mU/L.

**Table 2:** Molecular findings and the frequency of different somatic pathogenic variants found in patients with aldosterone-producing adenoma

Gene	Pathogenic Variant	N	Frequency (%)
*KCNJ5*	p.Gly151Arg, p.Leu168Arg, p.Glu145Gln, p.Gly151_Tyr152del	10	31.2
*ATP1A1*	p.Leu104Arg	1	3.1
*ATP2B3*	p.Leu425_Val426del	1	3.1
*CLCN2*	p.Pro48Arg, p.Glu195Gln	2	6.2
Total		14	43.6

Studied cohort: n = 32 patients and 33 APA (one patient had bilateral APAs).

**Figure 3:**
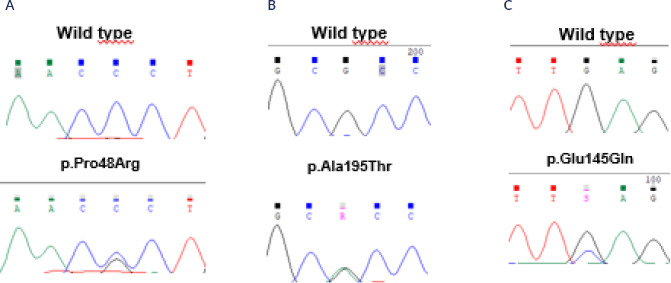
Electropherograms obtained by Sanger sequencing method from a patient with aldosterone-producing adenoma presenting the pathogenic variant (A) p.P48R and the pathogenic variants (**B**) p.Ala195Thr in the *CLCN2* gene, and (*C*) p.Glu145Gln in the *KCNJ5* gene.

**Figure 4:**
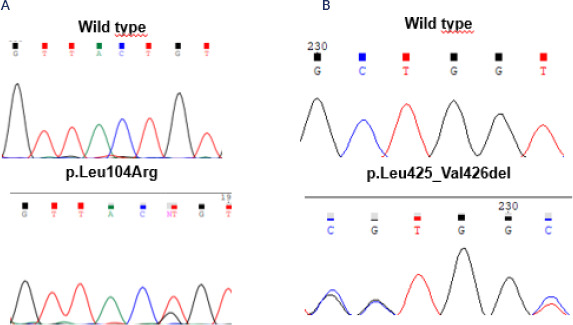
Electropherograms obtained by Sanger sequencing method of a normal control and (**A**) a patient with aldosterone-producing adenoma presenting the pathogenic p.L104R variant in the *ATP1A1* gene, and of a normal control and (**B**) a patient with aldosterone-producing adenoma presenting the pathogenic p.Leu425_Val426del variant in the *ATP2B3* gene.

In the *CLCN2* gene, we identified two PVs (6.25%): one patient (3.2%) carrying p.Pro48Arg (**Figure 3A**), and another (3.2%) with p.Ala195Thr (**Figure 3B**). We also observed the presence of the p.Glu145Gln PV in the *KCNJ5* gene in the same APA from this latter patient (**Figure 2C**). Two PVs were found in ATPase genes: p.Leu104Arg in *ATP1A1* (3.2%) and p.Leu425_Val426del (3.2%) in *ATP2B3* (*Figures 4A* and *4B*). No PVs were detected in the *CACNA1D*, \*GNAQ*, \*GNA11*, and *CTNNB1 *genes*.*

Patients with PVs in the *KCNJ5 *gene were predominantly female (90% *vs.* 45.45%; \*p* = 0.01) and were diagnosed with PA at a younger age (38 *vs*. 54 years; \*p* = 0.04), with a lower incidence of electrocardiogram abnormalities (20% *vs.* 59%; \*p* = 0.04). No significant differences were observed in body mass index, age at hypertension onset, defined daily doses of antihypertensives, family history of hypertension or early cardiovascular disease, mean arterial pressure, preoperative levels of aldosterone, renin, potassium, creatinine, or electrocardiogram changes, nodule size, or postoperative levels of antihypertensives and creatinine.

**Table 1** also lists the comparisons between clinical and laboratory data between patients with and without PVs in the *KCNJ5*, \*ATP1A1*, \*ATP2B3*, and \*CLCN2* genes. Patients with PVs exhibited an earlier onset of hypertension (38 *vs.* 55 years; \*p* = 0.02) and a trend toward a higher predominance of females (77% *vs.* 23%; \*p* = 0.09). Other parameters did not demonstrate significant differences between these groups.

For predicting clinical outcomes post-adrenalectomy in unilateral APA patients, the numerical PASO score was utilized in 24 of the 32 patients. Among these, 9 patients had complete clinical cure and 15 had partial clinical cure criteria. Comparing patients with and without PVs in genes related to APA pathogenesis, we observed that patients carrying PVs had higher rate of complete clinical cure (66.7% *vs.* 33.3%; \*p* = 0.05). Twenty-one patients experienced a complete biochemical cure, and three had a partial biochemical cure, showing no significant difference between patients with or without PVs (42.9% *vs.* 57.1%; \*p* = 0.71).

## DISCUSSION

Our findings evaluating a cohort diagnosed with APA revealed PVs in genes responsible for intracellular ionic homeostasis and cellular membrane potential, particularly in the *KCNJ5*. Additionally, we identified PVs in these genes, which appear to be unique to the Brazilian population. The study also confirmed an association between the presence of PVs and both female patients and younger ages at the time of APA diagnosis.

Our cohort, examined from both clinical and biochemical perspectives, did not significantly differ from other cohorts, either in Brazil or globally. Notably, among our patients, one presented with a bilateral lesion (3.1%). The prevalence of bilateral lesions in PA is not well-defined; however, a study of 164 patients reported seven cases of bilateral lesions, yielding a prevalence of 4.3% (^29,30^), which aligns with our findings. Another significant clinical aspect is the delayed diagnosis of PA, attributed to its lack of recognition by physicians (^2^) and the high heterogeneity among centers worldwide (^31^). Our data, consistent with previously published Brazilian studies, indicate a higher incidence of hypokalemia (84%) at diagnosis, suggesting severe hyperaldosteronism likely due to the prolonged interval between the onset of hypertension and PA diagnosis (^10,27^). This underscores the importance of educating both patients and healthcare professionals about PA to ensure timely diagnosis and intervention, thereby minimizing associated morbidity and mortality.

Somatic PVs were detected in genes associated with APA pathogenesis in 43.7% of our patients. Mutations in the *KCNJ5* gene, encoding the GIRK4 K^⁺^channel, were the most prevalent, accounting for 31.2%. These findings are consistent with averages reported in key studies. The first, based on the European Network for the Study of Adrenal Tumors (ENSAT) cohort, found somatic PVs in 54% of tumors, with *KCNJ5 *being the most affected gene (38%) (^31^). The second, a multicenter study, reported a prevalence of approximately 34% (^32^). Additionally, a single Brazilian study noted a prevalence of approximately 55% of PVs in genes associated with APAs, with *KCNJ5* being the most common (45%) (^27^).

An association was observed between pathogenic *KCNJ5* variants and a higher prevalence in female patients, an earlier PA diagnosis, fewer electrocardiographic alterations, and a trend towards earlier hypertension onset, echoing findings from a previous systematic review (^18^). Prior studies have demonstrated that somatic PVs in the *KCNJ5 *gene are prevalent in APAs, particularly among women and Asian populations (^33^), suggesting a sex-specific predisposition to certain genetic variants influencing aldosterone production in APAs (^34^).

In our cohort, the most frequently identified *KCNJ5 *mutations were G151R and L168R, consistent with prior studies. G151R mutations are more prevalent in Asian populations, while L168R mutations are more commonly reported in Western cohorts (^21,27,31,33^). We identified the *KCNJ5 *variant p.Gly151_Tyr152del, previously described (^35^) but not in the Brazilian population, resulting in a deletion of two amino acids, glycine 151 and tyrosine 152. This deletion, not affecting the downstream reading frame, is predicted by the Mutation T@ster 2025 online tool (https://www.genecascade.org/MutationTaster2025/modperl/MutationTaster.cgi) to be deleterious. The residues G151 and Y152 are highly conserved across species. G151, the first glycine in the GYG motif of the K^⁺^channel’s selectivity filter in KCNJ5, has been frequently affected in APAs. Functional studies indicate that substituting glycine 151 with arginine disrupts channel selectivity, leading to a positive shift in the reversal potential (^20^). Thus, the loss of glycine 151 and tyrosine 152, two of the three amino acids in the highly conserved GYG motif observed in the APA from our patient, is likely to severely impair the K*⁺*channel’s selectivity filter.

ATPases, expressed in adrenal cortex cells, regulate the homeostasis of Na^+^, K^+^, and Ca^2+^ ions. The genes *ATP1A1* and *ATP2B3* encode Na^+^/K^+^-ATPase 1 and Ca^2+^-ATPase 3, respectively. A PV in the *ATP1A1* gene was identified at a frequency of 3.1%, which is comparable to the 5.3% frequency observed in the ENSAT cohort of APAs (^36^). In the only Brazilian study published, the prevalence of the *ATP1A1* PVs was 3.2%, which is identical to our findings (^27^). A pathogenic *ATP2B3 *variant, which reduces Ca^2+^-ATPase pump activity, leading to increased intracellular Ca^2+^ and subsequent aldosterone production, was observed at a frequency of 3.1%. This is slightly higher than the 1.6% described in the ENSAT cohort (^36^) and in the Brazilian study (^27^), thereby confirming the rarity of PVs in this gene.

In this study, we identified two somatic variants in the *CLCN2 *gene, which encodes the voltage-dependent chloride channel Clc-2: p.Ala195Thr and p.Pro48Arg, with a frequency of 6.2%. Unlike our findings, pathogenic \*CLCN2* variants associated with APAs have not been previously described in either the ENSAT cohort or earlier Brazilian studies. Naturally occurring rare germline variants of the *CLCN2* gene have been reported in human populations, as detailed in European epilepsy cohorts (^36,37^), a Central African cohort (^38^), and they are also associated with leukoencephalopathy and familial hyperaldosteronism type II (^22,39^). The p.Ala195Thr variant is listed in ClinVar as of uncertain significance and has only been observed in the context of epileptic encephalopathy. No functional studies or associations with aldosterone-related phenotypes have been reported; thus, this study is the first to suggest a potential association between p.Ala195Thr and APAs.

The P48R germline variant in *CLCN2* has been rarely reported in individuals with primary aldosteronism (^34^); our study is the first to report an association of a somatic P48R variant with APA. Notably, our patient presented with both germline and somatic P48R variants. Although the proline at residue 48 is highly conserved across species, most *in silico* prediction tools classify this substitution as benign or of uncertain significance. In a study by Paul and cols. (^38^), six low-frequency missense variants in *CLCN2*, including P48R, R68H, G199A, R646Q, R725W, and R747H, found in a Central African population were analyzed for their functional impact on ClC-2 channel gating kinetics.

The P48R variant, located in the N-terminal cytoplasmic region, was functionally expressed in Xenopus laevis oocytes, and chloride currents were recorded using the two-electrode voltage-clamp technique. While some variants (R68H, R725W, and R747H) showed significantly accelerated voltage-dependent activation and enhanced steady-state Cl^⁻^ currents, others (*e.g.*, P48R) were associated with reduced current amplitudes, likely due to impaired trafficking or decreased membrane expression of the mutant channels. The authors proposed that N-terminal mutations might interfere with interactions between ClC-2 and proteins responsible for membrane trafficking or stabilization (^38^). This suggested mechanism differs from that proposed by Göppner and cols. (^39^), who studied a knock-in mouse model expressing equivalent human \*CLCN2* germline gain-of-function mutations previously identified in patients with early-onset primary aldosteronism. Their findings indicated that these mutations abolished the voltage-dependent closure of ClC-2 channels, resulting in persistent chloride efflux (^39^). Collectively, these studies support a potential pathogenic role for \*CLCN2* variants, such as the one identified in our study, although further investigations are needed to clarify their clinical relevance and the underlying molecular mechanisms involved.

The *CACNA1D* gene encodes the Cav3.2 T-type voltage-dependent calcium channels, which regulate Ca^2+^ influx in adrenal glomerulosa cells. We did not identify any *CACNA1D* variants. Conversely, a distinct Brazilian cohort reported a low prevalence of 1.6%. European studies have indicated an average prevalence of 9.3%, varying by 0%-13.6% (^31^). Scholl and cols. (^37^) observed a similar prevalence of 10.3%. However, a multicenter study by Nanba and Rainey (^34^) reported a frequency of 21%, primarily in Black populations. These findings suggest that the prevalence of *CACNA1D* variants may vary significantly across populations, with Brazilians exhibiting a lower frequency.

Pathogenic *CTNNB1* gene variants result in abnormal activation of the Wnt pathway, blocking β-catenin phosphorylation and degradation. This process leads to the activation of the adrenal gland (^40^). No *CTNNB1* variants were identified in this study. Another Brazilian cohort found *CTNNB1* variants in 3.2% of cases (^27^), similar to the 2.1% prevalence reported by Scholl and cols. (^37^) and 5.1% reported by Åkerström and cols. (^25^). This study and other Brazilian series did not identify variants in the *GNA11* or *GNAQ*, genes encoding Gα subunits G11 and GQ, respectively. *GNA11* and \*GNAQ* have been implicated as co-driver genes in APAs. Somatic mutations in these genes may inhibit GTP hydrolysis, resulting in a constitutively active GTP-bound state of Gα. This change sustains phospholipase Cβ signaling, extends intracellular Ca²^⁺^mobilization, and ultimately promotes autonomous aldosterone production (^40^). Although rare, double mutations involving *CTNNB1* and either \*KCNJ5* or *CACNA1D* have been identified in aldosteronomas (^24-26^). Recent evidence indicates that *CTNNB1* variants may co-occur with *GNA11* or *GNAQ* mutations in a significant subset (~59%) of *CTNNB1*-mutant APAs presenting during puberty, pregnancy, or menopause. This co-occurrence correlates with specific clinical, radiological, and pathological features (^26,37^).

Extended molecular characterization of *CTNNB1*-mutant APAs with *GNA11/GNAQ* mutations has revealed unique genotype and phenotype signatures. These include upregulated LHCGR, modified CYP11B2/B1 expression profiles, and hyperplasia in adjacent adrenal tissue (^26^). No isolated *GNA11* or *GNAQ* mutations have been shown to independently cause APA formation; in neighboring hyperplastic zona glomerulosa, these mutations are silent and presumably require a \*CTNNB1*-driven proliferative context to become pathogenic (^26^). *CYP11B2* immunohistochemistry (IHC) is a valuable tool for guiding genetic testing. It significantly increases the detection rate of somatic genetic drivers in PA by specifically identifying functional aldosterone-producing cell clusters or nodules (^35,41^). These may be small or mixed with non-functioning tissue and thus overlooked by conventional gross dissection.

Next-generation sequencing (NGS) enhances the detection of mutations in adrenal samples negative by Sanger sequencing. Using CYP11B2 IHC to target the probable origin of aldosterone production alongside NGS results in a higher overall prevalence of somatic APA variants (85%) compared to conventional methods (50%). Caroccia and cols. (^42^) examined 127 adrenal samples from patients with APA using double IHC for CYP11B1 and CYP11B2, followed by Sanger and NGS. Three IHC patterns were identified: CYP11B2-positive adenomas, mixed CYP11B1/CYP11B2 adenomas, and multiple small CYP11B2-positive nodules. The incidence of *KCNJ5* mutations varied significantly by IHC pattern, with the highest frequency observed in mixed CYP11B1/CYP11B2 tumors. While Sanger sequencing detected *KCNJ5* mutations in 44% of samples, NGS identified an additional 10% of samples negative by Sanger sequencing. These findings suggest that IHC-guided sequencing focused only on CYP11B2-positive areas may also overlook critical mutations and also highlight the broader utility of NGS (^42^). Goldbaum and cols. (^43^) also found that IHC can aid in assessing outcomes in patients surgically treated for APA. They found that classical histological patterns serve as independent predictors of more severe primary aldosteronism and are associated with both complete biochemical and clinical remission in these patients (^43^).

A key limitation of our study is the exclusive reliance on Sanger sequencing, which targeted only hotspot exons of the selected genes. The absence of IHC-guided analysis and/or NGS in Sanger-negative samples likely contributed to an underdetection of mutations associated with APAs. In conclusion, our findings emphasize the established phenotype-genotype correlation when genes regulating intracellular ionic homeostasis and cellular membrane potential are implicated in APA pathogenesis. A recent study demonstrated that the Brazilian population is characterized by a mix of Indigenous American, European, and African ancestries, uncovering over 8 million previously unidentified variants (^44^). Thus, identifying PVs in this study enhances our understanding of the genetic landscape in Brazilian patients with primary aldosteronism.

## Data Availability

data generated during and/or analyzed during the study are available from the corresponding author upon request.
